# *Announcement*: American Heart Month — February 2017

**DOI:** 10.15585/mmwr.mm6606a6

**Published:** 2017-02-17

Each February, the observance of American Heart Month helps raise awareness of ways to stay heart healthy and prevent heart disease. For 30 years, the number of deaths from heart disease, the number one killer of persons in the United States, declined. However, that progress has stalled in recent years, potentially because of high rates of obesity and hypertension, important risk factors for heart disease (*1*). With increased awareness and education, everyone can work together to prevent the conditions that lead to heart disease.

Heart disease is responsible for one in every four deaths (>630,000) in the United States each year. Approximately 790,000 men and women have a heart attack each year (*2*,*3*). Conditions such as obesity and physical inactivity, and behaviors such as consuming an unhealthy diet or using tobacco, are major contributors to heart disease and heart attacks (*1*). Approximately two thirds of adults are overweight or have obesity (*4*). Obesity can lead to high blood pressure, blood glucose problems (including diabetes), and trouble sleeping, all of which strain the heart (*5*). In addition, just one in five adults meets the current federal recommendations for 150 minutes of moderate activity each week and muscle strengthening two times per week (*6*).

In observance of American Heart Month 2017, CDC is encouraging everyone to have a heart-to-heart with their loved ones and plan to make small changes to their diet and behavior to prevent heart disease. Many of the conditions that contribute to heart disease have a genetic component, so learning a family’s health history is an important step toward recognizing a person’s risk for developing heart disease. Quitting smoking, avoiding secondhand smoke, eating foods low in sodium and trans fats, and getting appropriate amounts of physical activity are ways families can incorporate heart-healthy behaviors into their lives.

CDC has additional resources to help families improve their heart health at http://millionhearts.hhs.gov/news-media/events/heart-month.html.
